# Impact of failed ab-interno trabeculectomy (trabectome) on subsequent XEN45 gel stent implantation in pseudophakic eyes

**DOI:** 10.1007/s10792-021-01977-w

**Published:** 2021-08-07

**Authors:** D. Kiessling, C. Rennings, M. Hild, A. Lappas, T. S. Dietlein, G. F. Roessler, Randolf Alexander Widder

**Affiliations:** 1 Department of Ophthalmology, St. Martinus-Krankenhaus, Gladbacher Str.26, 40219 Düsseldorf, Germany; 2 Department of Ophthalmology, University Hospital of Cologne, Kerpener Str 62, 50935 Cologne, Germany; 3 Department of Ophthalmology, RWTH Aachen University, Pauwelstr. 30, 52074 Aachen, Germany

**Keywords:** Glaucoma incisional surgery, MIGS, XEN, Microstent, Trabectome, Open angle glaucoma

## Abstract

**Purpose:**

To determine the impact of failed ab-interno trabeculectomy on the postoperative outcome of subsequent XEN45 gel stent (Allergan, CA, USA) implantation in pseudophakic eyes.

**Methods:**

In this retrospective single-center study, we included 60 pseudophakic eyes from 60 participants who underwent XEN45 gel stent implantation. Thirty eyes each underwent primary stent implantation (control group) or had previously undergone a failed ab-interno trabeculectomy (trabectome group). The groups were matched at a 1:1 ratio based on the following criteria: preoperative and maximum Intraocular pressure (IOP), preoperative medication score, cup/disk-ratio, follow-up time, best-corrected visual acuity at baseline, age, and the proportion of patients classified as primary open angle glaucoma or exfoliation glaucoma. We defined a successful surgery by the following three scores: an IOP reduction > 20% and IOP at the longest follow-up < 21 mmHg (Score A) or < 18 mmHg (Score B) or IOP ≤ 15 mmHg and an IOP reduction ≥ 40% (Score C). One open conjunctival revision was allowed in all scores, and a repeat surgery was considered a failure.

**Results:**

Following an average follow-up period of 22 ± 12 months, we observed a mean IOP reduction of 38%, from 23.5 ± 5.2–14.5 ± 5.0 mmHg. Comparative analyses between the groups did not reveal a significant difference in the postoperative IOP, postoperative medication score, side effects, revision rate, repeat surgery rate, or success rate.

**Conclusions:**

Trabectome is a viable first-line procedure for medically uncontrolled glaucoma before filtering ab-interno microstent surgery is considered.

## Introduction

In addition to established procedures that are used to treat uncontrolled glaucoma, Microinvasive glaucoma surgery (MIGS), which targets the iridocorneal angle, has been used in routine clinical practice in recent years. trabectome surgery (Microsurgical Technology, Redmond, WA, USA), being just one of them, pursues an ab-interno approach. Thus, it does not require the penetration of the conjunctiva. It facilitates an electroablation of the trabecular meshwork and improves access to the physiological system of aqueous outflow through the Schlemm’s canal [1–8].

Jea et al. [9] postulated that Trabectome surgery is a reasonable choice of primary surgery to treat glaucoma and should be considered before traditional filtering surgery. They demonstrated that in terms of success rates and complications, there is no disadvantage in performing trabeculectomy after a failed trabectome surgery.

In addition, the spectrum of filtering surgery is enhanced through MIGS procedures as well. XEN45 gel stent implantation (Allergan, Dublin, CA, USA) is conducted ab-interno via the anterior chamber through the trabecular meshwork and the scleral wall. Moreover, it targets the formation of a subconjunctival bleb. The implant has a small lumen, which assures a moderate drainage of the aqueous humor into the subconjunctival space with minimal side effects, such as hypotony. Several researchers have reported on their favorable safety profile, along with the promising IOP-lowering potential [10–16].

More recently, a comparative analysis between pseudophakic eyes that underwent prior trabeculectomy and a matched pseudophakic control group demonstrated that a failed trabeculectomy does not affect the outcome of XEN gel stent implantation [17]. However, there is no evidence for the viability of the aforementioned procedure after a failed ab-interno trabeculectomy. Therefore, we compared the outcomes of XEN45 gel stent implantation of two matched groups comprising patients with pseudophakia who had either undergone a previous failed trabectome surgery or had undergone primary stent implantation.

## Methods

### Study design and patients

This single-center retrospective study was based on the data acquired from the Department of Ophthalmology, St. Martinus-Krankenhaus Düsseldorf, Germany. The retrospective study protocol and data accumulation were conducted with the approval of the Institutional Review Board (Ethik und Kommission Klinische Studien, Dernbacher Gruppe Katharina Kasper, Germany). We adhered to all tenets of the declaration of Helsinki. For the Trabectome group, we reviewed our database and identified patients who underwent secondary stent implantation after a failed ab-interno trabeculectomy from 2015–2020. We excluded patients with a history of previous eye surgery, including trabeculectomy, deep sclerectomy, iStent inject implantation (Glaukos, CA, USA), cyclophotocoagulation, vitrectomy, canaloplasty, and selective laser trabeculoplasty. We also excluded those with a follow-up < 6 months. Performing trabectome surgery mostly as a combined procedure the majority of patients receiving XEN stent surgery after trabecome surgery were pseudophakic. Therefore, the small group of phakic patients who received secondary XEN Gel Stent implantation were excluded from the study to maintain group homogeneity.

The inclusion criteria for the control group were as follows: (i) no history of previous eye surgery, other than cataract surgery, (ii) absence of concomitant eye disease, such as neovascular glaucoma and uveitis, and (iii) follow-up data of at least 6 months.

The eligible patients were matched with those who had undergone ab-interno trabeculectomy at a 1:1 ratio, based on the following criteria: preoperative IOP, maximum known preoperative IOP, preoperative medication score, cup/disk-ratio, follow-up time, best-corrected visual acuity (BCVA) at baseline, and age. Moreover, we matched the proportion of patients classified as Exfoliation glaucoma (XFG) between the groups. If both eyes of a patient were eligible, we included the eye with a longer follow-up.

The IOP was assessed by Goldmann applanation tonometry. We collected a maximum of three IOP measurements, prior to the surgery, and averaged them to evaluate the baseline IOP. The medication score comprised the number of IOP—lowering medication classes administered at baseline and follow-up. The BCVA was measured using standard Snellen charts.

### Surgical technique

XEN45 gel stent implantation was performed according to procedures described in previous studies [10, 17]. Initially, mitomycin C (0.1 mg/mL) was injected under the conjunctiva of the upper nasal quadrant, at a distance of 6 mm from the limbus. Following a temporal paracentesis and paracentesis at the 5 or 7 o’clock position, and anterior chamber stabilization using viscoelastic substance, the stent was placed via its injector device. The apex of the injector was driven through the trabecular meshwork and sclera, at a distance of 3 mm from the limbus. The stent was then injected under the conjunctiva, and the injector was removed. The position of the stent was confirmed via gonioscopy. Moreover, the viscoelastic substance was removed from the anterior chamber by irrigation. The aim of surgery was to regulate the IOP without using anti-glaucomatous medications.

Therefore, all patients, without sufficiently reduced postoperative IOP, underwent surgical revision. Instead of needling, the patients were treated via an open conjunctival approach [10, 18]. Following the incision of the conjunctiva at the limbus, the stent was prepared. Following the removal of the scar tissues, the conjunctiva was refixated at the limbus with two absorbable 9.0 sutures.

The patients were subjected to a standardized postoperative topical therapy, including an antibiotic ointment (Floxal AS, Bausch & Lomb, Frankfurt, Germany) and steroid ointment (Ultracortenol AS, Agepha, Senec, Slovakia) thrice a day, the dose was tapered over 4 weeks. Previously prescribed topical anti-glaucomatous medications were discontinued.

### Outcome measurement

Changes in the IOP and medication scores at the longest follow-up examination were the primary clinical endpoints. These outcomes were further defined as success or failure by three separate scores. According to scores A and B, an absolute IOP < 21 mmHg or < 18 mmHg at the follow-up examination and a postoperative IOP reduction > 20%, respectively, qualified for success. The aforementioned scores were chosen based on the tube versus trabeculectomy study [19]. In contrast, the criteria for Score C were an absolute IOP ≤ 15 mmHg and a postoperative IOP reduction ≥ 40%, based on the criteria of the World Glaucoma Association [20]. In all scores, one open conjunctival revision was allowed. Repeat surgeries, including an additional glaucoma surgery other than open conjunctival revision was considered a failure in all scores.

### Statistical analyses

We performed the statistical analyses using SPSS (Version 24.0, IBM Corp. Armonk, NY, USA) and the statistical programming language R V3.2.2 (R Foundation for Statistical Computing, Vienna, Austria). We compared the outcome measurements between the trabectome group and the control group via a Mann–Whitney U-test and Fisher**’**s exact test. Moreover, we conducted a log-rank test and visualized the differences using Kaplan–Meier curves. The threshold for statistical significance was defined as *p* < 0.05.

## Results

We identified a total of 751 eyes of 581 patients, with 81 (11%) patients undergoing secondary XEN45 gel stent implantation after a failed ab-interno trabeculectomy. Of these patients, 76 (94%) and five (6%) patients were pseudophakic and phakic, respectively. We eventually retained 30 eyes of 30 patients in the trabectome group, of which 16 (53%) eyes were classified as Primary open angle glaucoma (POAG), 14 (47%) were classified as XFG. Standalone Trabectome surgery and combined surgery with phacoemulsification or phacoemulsification and trabecular aspiration surgery were performed in 11 (37%), 13 (43%), and six eyes (20%), respectively [21–23]. The mean latency between primary ab-interno trabeculectomy and XEN Gel stent implantation was 3.2 ± 1.9 years.

Of the 380 pseudophakic eyes in the control group, 108 eyes of 108 patients met the inclusion criteria. After matching 30 eyes of 30 patients were included in the control group, with proportions of eyes with POAG and XFG similar to those in the Trabectome group. Table 1 summarizes the baseline data.

**Table 1:** Baseline data of patients

	Trabectome group *n* = 30	Control group *n* = 30	*p*-value
Age (years)	77.2 ± 7	77.8 ± 7	0.74
Caucasian	30/30	30/30	> 0.99
Maximum preoperative IOP (mmHg)	31.3 ± 6.4	31.6 ± 8.0	0.87
Actual preoperative IOP (mmHg)	23.5 ± 4.9	23.5 ± 5.6	0.96
Medication score initial	2.3 ± 1.0	2.7 ± 1.1	0.19
Exfoliation glaucoma (n)	14/30	14/30	> 0.99
Baseline C/D ratio	0.8 ± 0.2	0.8 ± 0.2	0.39
Follow-up time (months)	22.6 ± 14	21.5 ± 10	0.71
Baseline BCVA (logMAR)	0.24 ± 0.22	0.33 ± 0.33	0.23

IOP: Intraocular pressure; C/D: Cup/disk; BCVA: Best-corrected visual acuity and logMAR; Logarithm of the minimum angle of resolution

Following an average follow-up of 22 ± 12 months, we observed a mean IOP reduction of 38%, from 23.5 ± 5.2–14.5 ± 5.0 mmHg. Moreover, the mean medication score reduced by 84%, from 2.5 ± 1.1–0.4 ± 0.8 mmHg in the entire cohort.

The comparative analysis between the trabectome and control groups did not reveal any significant difference in the postoperative IOP, postoperative medication score, revision rate, repeat surgery rate, or success rate (Table 2).

**Table 2:** Intraocular pressure and medication scores at baseline and the longest follow-up

	All *n* = 60	Trabectome group *n* = 30	Control group *n* = 30	*p*-value
IOP preoperative (mmHg)	23.5 ± 5.2	23.5 ± 4.9	23.5 ± 5.6	0.96
IOP postoperative (mmHg)	14.5 ± 5.0	14.7 ± 5.1	14.3 ± 5.0	0.72
Medication score preoperative	2.5 ± 1.1	2.3 ± 1.0	2.7 ± 1.1	0.19
Medication score postoperative	0.4 ± 0.8	0.3 ± 0.8	0.5 ± 0.9	0.53
Revision n (%)	25/60 (42%)	11/30 (37%)	14/30 (47%)	0.44
Repeat surgery n (%)	7/60 (12%)	3/30 (10%)	4/30 (13%)	0.69

IOP: Intraocular pressure

Side effects in the trabectome and control groups included self-limited intraoperative subconjunctival bleeding and choroidal effusion because of transient hypotony in one and two patients, respectively. There were no severe side effects, such as retinal detachment, leakage, blebitis, and endophthalmitis, in either groups (Table 3).

**Table 3:** Complications

	Trabectome group *n* = 30	Control group *n* = 30	*p*-value
Intraoperative subconjunctival bleeding	1	0	0.32
Chorioidal effusion	0	2	0.16
Macular edema, retinal detachment, leakage, blebitis, endophthalmitis	0	0	> 0.99

**Fig. 1:**
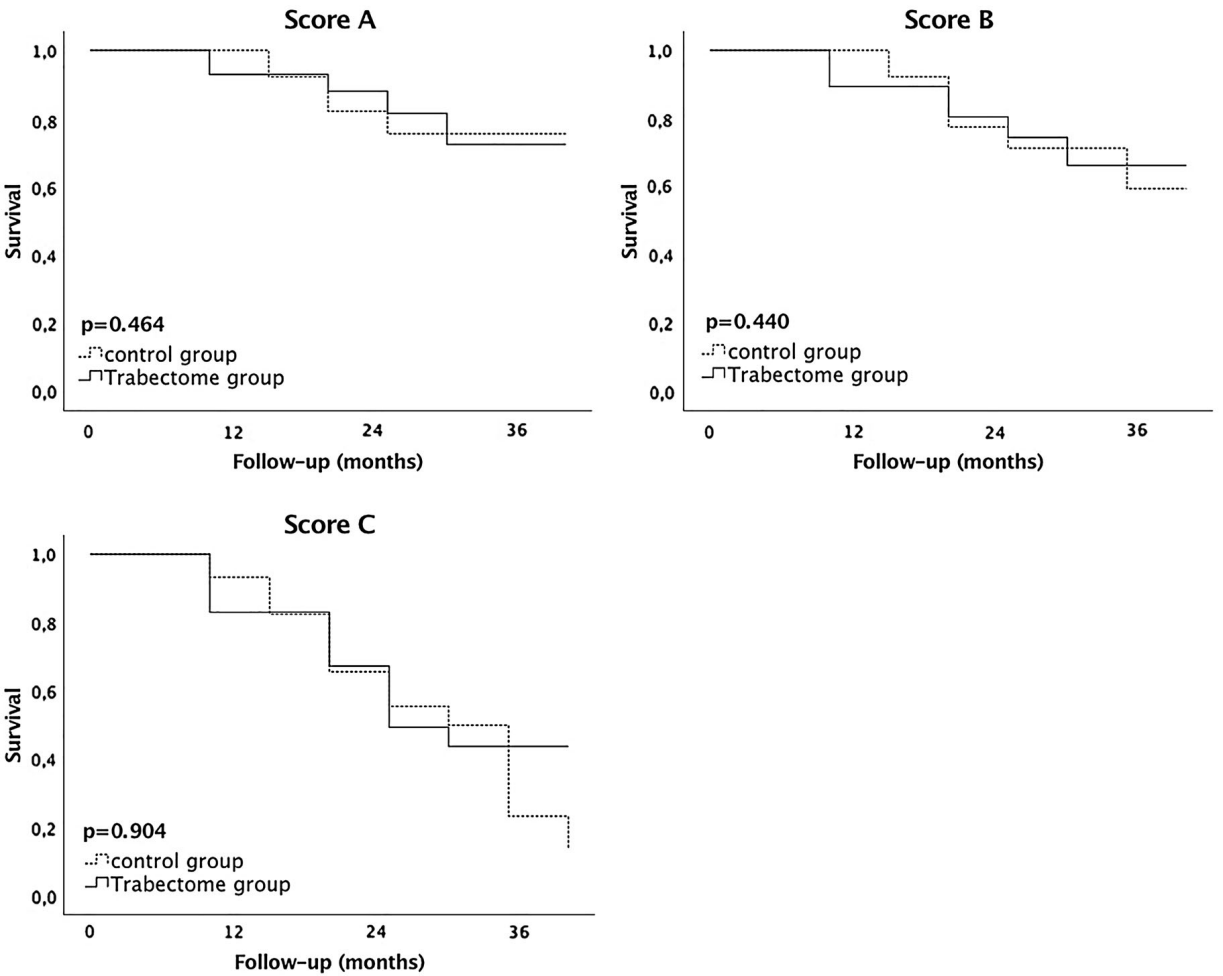
Kaplan–Meier survival curves comparing success rates in the control group and the Trabectome group following XEN45 Gel Stent implantation. Score A: The intraocular pressure (IOP) at follow-up is < 21 mmHg, IOP reduction is > 20%, one revision surgery is allowed, and no repeat surgeries. Score B: The IOP at follow-up is < 18 mmHg, IOP reduction is > 20%, one revision surgery allowed, and no repeat surgeries. Score C: The IOP at follow-up is ≤ 15 mmHg, IOP reduction is ≥ 40%, one revision surgery allowed, and no repeat surgeries

The Kaplan–Meier curves showing the comparison of success rates between the two groups according to the applied scores displayed corresponding trends, which remained parallel throughout (Fig. 1).

## Discussion

We found that a failed trabectome surgery does not affect the outcomes, including postoperative IOP, postoperative medication score, side effects, revision rate, repeat surgery rate, and success rate, of a subsequent XEN45 gel stent implantation. Our findings, which were consistent with those of previous studies, provided further evidence for the efficacy of XEN gel stent implantation in reducing both IOP and medication scores while retaining a favorable safety profile [10–16].

Our findings confirm that ab-interno surgery in the iridocorneal angle does not interfere with the outcome of a subsequent filtering surgery, affirming the results of the study by Jea et al. [9] This could be attributed to the fact that trabectome surgery does not require the penetration of the conjunctiva [9]. It was postulated that previous conjunctival incisional surgery leads to an increased proliferation of fibroblasts and inflammatory cells of the conjunctiva, which contributes to a higher probability of filtering bleb failure as a result of postoperative inflammation [24]. Our results suggest that, by sparing the conjunctiva, Trabectome surgery allows a more predictable response of IOP after secondary filtering surgery. We conclude that sufficient subconjuctival aqueous drainage can succeed independently from trabecular meshwork excision procedures, which have been carried out previously ab-interno.

To date, there have been no investigations on the histopathological properties of a failed trabectome surgery. Nonetheless, a recent histological examination has investigated two cases with insufficient outcomes after non-ablative ab-interno trabeculotomy using specially designed microhooks [25]. Tsutsui et al. postulated that IOP reduction via microhook ab-interno trabeculotomy occurs either due to a decrease in the resistance of the trabecular meshwork or an addition of an unconventional aqueous outflow path. The authors determined the tissue response via immunohistochemistry and found it not extend beyond the Schlemm’s canal and the collector channels toward structures on the ocular surface.

Previous surgeries, such as trabeculectomy, cataract surgery, vitrectomy, and keratoplasty, increase susceptibility to subconjunctival scarring. This in turn is of crucial importance for the long-term effectiveness of the subsequent filtering surgery, including XEN Gel Stent implantation [26]. However, following prior trabeculectomy, the outcomes of XEN Gel Stent implantation are not inferior to that of primary stent implantation [17]. We validated a similar trend in post-trabectome eyes, which were compared to those that underwent primary XEN Gel Stent implantation.

Among our entire cohort, there was a mean IOP reduction of 38%, a postoperative IOP of 14.5 ± 5.0 mmHg, and a postoperative medication score of 0.4 ± 0.8. While 42% of the patients underwent an open conjunctival revision surgery, 12% underwent a repeat surgery. Our results are concordant with those of previous studies on efficacy of XEN gel stent implantation [10, 11, 27, 28].

The retrospective study design and limited sample size were the major limitations of our study. Additionally, eyes that had undergone trabectome surgery prior to XEN gel stent implantation might have not been initially considered suitable for primary stent implantation. This possibly introduced a selection bias. However, it did not generate inferior results. Therefore, we hypothesized that this did not have a considerable impact on the study findings.

We validated that a failed trabectome surgery does not affect the outcome of subsequent XEN45 gel stent implantation. Therefore, we believe that trabectome surgery is a viable first-line procedure for medically uncontrolled glaucoma before filtering ab-interno microstent surgery is considered.

Despite little or no evidence, we assume that our findings might be applicable to other trabecular meshwork excision procedures, such as goniotome (Microsurgical Technology, Redmond, WA, USA) or Kahook Dual Blade surgery (KDB, New World Medical, Rancho Cucamonga, CA).
